# Electrophysiological Correlates of Shyness Affected by Facial Attractiveness

**DOI:** 10.3389/fpsyg.2021.739585

**Published:** 2022-01-05

**Authors:** Xiaofan Xu, Bingbing Li, Ping Liu, Dan Li

**Affiliations:** Department of Psychology, School of Education, Shanghai Normal University, Shanghai, China

**Keywords:** EEG, delta-beta correlation, shyness, facial attractiveness, public speaking

## Abstract

Previous neurological studies of shyness have focused on the hemispheric asymmetry of alpha spectral power. To the best of our knowledge, few studies have focused on the interaction between different frequencies bands in the brain of shyness. Additionally, shy individuals are even shyer when confronted with a group of people they consider superior to them. This study aimed to reveal the neural basis of shy individuals using the delta-beta correlation. Further, it aimed to investigate the effect of evaluators’ facial attractiveness on the delta-beta correlation of shyness during the speech anticipation phase. We recorded electroencephalogram (EEG) activity of 94 participants during rest and anticipation of the public speaking phase. Moreover, during the speech anticipation phase, participants were presented with high or low facial attractiveness. The results showed that, as predicted, the delta-beta correlation in the frontal region was more robust for high shyness than for low shyness during the speech anticipation phase. However, no significant differences were observed in the delta-beta correlation during the baseline phase. Further exploration found that the delta-beta correlation was more robust for high facial attractiveness than low facial attractiveness in the high shyness group. However, no significant difference was found in the low-shyness group. This study suggests that a stronger delta-beta correlation might be the neural basis for shy individuals. Moreover, high facial attractiveness might enhance the delta-beta correlation of high shyness in anticipation of public speaking.

## Introduction

Shyness is a personality trait that is ubiquitous in interpersonal communication. It involves an excessive concern about negative evaluation and avoidance of participation in social situations that would otherwise be pleasurable or important to one’s professional or personal growth (Henderson and Zimbardo, 2001). Studying the underlying neural basis of shyness can help us better understand shyness and ultimately overcome it. Major electroencephalogram (EEG) studies on shyness have focused on hemispheric asymmetry of alpha (8–13 Hz) spectral power (e.g., Schmidt and Fox, 1994; Schmidt, 1999; Schmidt et al., 1999; Beaton et al., 2008; Jetha et al., 2009). The EEG alpha asymmetry in the frontal region of the brain is associated with the experience of approach-related and avoidance-related emotions (Davidson and Fox, 1982) and approach/withdrawal motivation (Fox and Davidson, 1987). Stronger left frontal EEG activation is associated with approach-related emotions and approach motivation, whereas stronger right frontal EEG activation is related to avoidance-related emotions and avoidance motivation (Sutton and Davidson, 1997; Harmon-Jones, 2003; Honk and Schutter, 2006). However, not all studies support this relationship between alpha asymmetry and affective/motivational, with some supporting it partially and with some disconfirmed it. Therefore, the relationship between alpha asymmetry and affective/motivational propensity remains unclear and needs to be discussed (Vecchio and De Pascalis, 2020). Some studies of frontal alpha asymmetry for shyness suggest that shy participants displayed relatively more significant right frontal EEG activity at rest (Schmidt, 1999; Beaton et al., 2008; Jetha et al., 2009). Other studies have also found an increase in alpha activity in the right, but not the left, frontal EEG during anticipation of public speaking in the shy children sample (Schmidt et al., 1999). However, few studies have focused on the interaction between different frequency bands in the brain of shyness, which is another vital aspect to consider in understanding the neural basis.

The brain oscillates over a wide range of frequencies, from fast to slow-wave activities (Miskovic et al., 2011). Different brain oscillations are associated with a range of cognitive processes, emotional states, and behaviors (Klimesch, 1999; Varela et al., 2001; Knyazev, 2007; De Pascalis et al., 2012). Cross-frequency coupling may represent a neural code that integrates the interactions of different frequency bands to facilitate the exchange of information between functionally different nervous systems (Friston, 1997; Varela et al., 2001). The delta frequency range are slow-wave oscillation that are hypothesized to stem from subcortical regions responsible for motivation, emotion, and reward processing. In comparison, beta frequency ranges are fast-wave oscillations that are hypothesized to stem from the cortex responsible for attentional control, cognitive processing, and regulation (Engel et al., 2001; Knyazev and Slobodskaya, 2003; Knyazev, 2012). The cross-frequency correlation between slow and fast-wave oscillations is hypothesized to reflect the cross-talk between cortical and subcortical brain regions (Schutter et al., 2006). Although cross-frequency coupling in the brain of shyness is still unclear, researchers have investigated it in social anxiety during resting and speech anticipation conditions (e.g., Harrewijn et al., 2016). Previous studies suggested that the enhanced delta-beta correlation reflect excessive neural regulation and has been associated with anxiety (Phelps et al., 2016; De Pascalis et al., 2020; Poole et al., 2020). For instance, Miskovic et al. (2010) found that individuals with high social anxiety showed a significantly stronger positive delta-beta correlation in the frontal region than a low socially anxious group while anticipating public speaking. Harrewijn et al. (2016) found that compared to low socially anxious individuals, high socially anxious individuals showed a significantly stronger negative delta-beta correlation in the frontal region. Harrewijn et al. (2018) also found a significantly stronger negative delta-beta correlation in the frontal region for (sub) clinical social anxiety disorder (SAD) during anticipation. It is believed that the negative correlation could still be explained by increased crosstalk between cortical and sub-cortical regions just in different direction (Harrewijn et al., 2016). No significant differences were found during the resting phase. Contrastingly, only Miskovic et al. (2011) found that the positive delta-beta correlation could be reduced by treating SAD during both resting and speech anticipation conditions. The findings of these studies are suggest that the delta-beta correlation as an electrocortical measure of social anxiety seems more promising during the anticipation of a socially stressful situation than in the resting state. SAD is a clinical disorder whereas shyness is a normative temperamental predisposition of being uncomfortable in social situations (Heiser et al., 2003). They are different in a number of ways (Heiser et al., 2003). For example, in general, shy individuals do not experience the degree of functional impairment which experienced among socially anxious individuals (Turner et al., 1990). However, previous literature suggested that shyness and SAD may also share several similar characteristics, including symptoms across somatic (e.g., trembling, sweating, blushing), cognitive (e.g., fear of negative evaluation by others), and behavioral (e.g., avoidance of social situations) (Turner et al., 1990). That is, individuals with shyness or SAD are all likely to avoid social situations and experience stress and anxiety. Therefore, we propose to reveal the neural basis of shy individuals through delta-beta correlation analysis based on the existing studies during resting and anticipatory speech states.

Shy people have negative self-evaluations and increased anxiety and avoidance in social situations (Carducci and Conkright, 2020), which can be even more serious when they faced with a group of people they think are superior to them (Crozier, 1982). Social comparison, or comparing oneself with others, is an important means of self-evaluation (Festinger, 1954). Previous studies suggested that upward social comparisons, or comparing oneself with others who possess more positive characteristics, might exacerbate negative self-evaluations associated with anxiety mood (Antony et al., 2005). Facial attractiveness is a pervasive factor in everyday life and plays a vital role in human society (Mitrovic et al., 2016). Research has shown that more attractive people are judged more positively in a wide range of aspects and are given preferential treatment in many areas of life, called an “attractiveness halo” (Langlois et al., 2000; Liang et al., 2010). For example, attractive people are perceived as more competent, have better social skills and have higher intelligence (Dion et al., 1972; Hamermesh and Biddle, 1993). That is, people always have positive stereotypes about attractive people (Langlois et al., 2000; Chelnokova et al., 2014). Therefore, high facial attractiveness might increase the negative self-evaluations of shy individuals and increase anxiety and avoidance in social situations (Crozier, 1982; Langlois et al., 2000; Chelnokova et al., 2014; Carducci and Conkright, 2020). The second goal of this study was to investigate further the effect of evaluators’ facial attractiveness on the delta-beta correlation of shyness during the speech anticipation stage.

To the best of our knowledge, at present, few studies have focused on the interaction between different frequencies bands in the brain of shyness. This study aimed to reveal the neural basis of shy individuals using the delta-beta correlation. We recorded EEG activity during rest and anticipation of the public speaking phase and analyzed the EEG delta-beta coupling pattern in both conditions. According to the theory of Gray and McNaughton (2000), delta-beta correlation occurrence might be state-dependent (Knyazev et al., 2006). Moreover, previous studies have found that socially anxious and (sub) clinical SAD individuals showed significantly stronger negative delta-beta correlations in the frontal region while anticipating public speaking (Harrewijn et al., 2016, 2018). Therefore, we predicted that the delta-beta correlation in the frontal region was stronger for high shyness than for low shyness during the speech anticipation phase but not during the baseline. This was accompanied by subjective reporting that high shyness was more significant associated with nervousness and avoidance. Furthermore, shy individuals are even shyer when confronted with a group of people they consider superior to them (Crozier, 1982). Therefore, we further explored the effect of facial attractiveness on the delta-beta correlation of shyness during the speech anticipation phase. We predicted a stronger delta-beta correlation for high facial attractiveness than low facial attractiveness for high shy individuals but not for low shy individuals.

## Materials and Methods

### Participants

A total of 837 undergraduate and graduate students from Shanghai Normal University (Shanghai, China) were screened using the Revised Cheek and Buss Shyness Scale (RCBS) (Cheek, 1983). The students completed RCBS in psychology courses and online, and received gifts for their participation. Students with scores in the upper and lower thirds of the sample were contacted by telephone to determine their interest in participating in the formal study (Schmidt and Fox, 1994), that is, the score of the low shyness group was less than 31 and high shyness group was more than 40. A total of 126 students agreed to participate in the follow-up study: 58 high shyness (22 males, 36 females) and 68 low shyness (25 males, 43 females). Participants were asked to complete the RCBS two times, that is to say, the first measurement was at screening phase and the second measurement was before the experiment, which confirm whether they were still high or low shyness. According to the cut-offs of first screening, if they were still high or low shyness in the second measurement, they were consistent participants, otherwise, they were inconsistent participants. And then we exclude inconsistent participants (*n* = 26). Additionally, six subjects were excluded due to left-handedness (*n* = 5), assessed using the Edinburgh handedness inventory (Oldfield, 1971), and data acquisition problems (*n* = 1). The final sample of 42 high shyness (29 females, mean age = 21.33, *SD* = 2.03) and 52 low shyness (34 females, mean age = 21.77, *SD* = 1.85) were used for data analysis. The two groups did not differ in age, *t*(92) = –1.09, *p* = 0.28, and gender distribution χ^2^(1) = 0.14, *p* = 0.71. Participants who reported they had a history of brain injury, a history of psychiatric disorder including SAD were excluded from the study. In the current study, none of the subjects reported the above. Thus, all participants were included. All participants had a normal or corrected-to-normal vision. Participants were paid for their participation.

### Materials

#### Facial Photos

Facial photos of 193 college-age Chinese people (96 males and 97 females) were collected from the Internet. The criteria of selection for faces were head position forward, direct gaze, neutral expression, and no celebrities. First, the face images were standardized using Photoshop CC. All images, which only keep the face, hair, and part of the neck, were grayscale faces on a white background and adjusted to equal size, brightness, and contrast. Then, 25 undergraduate and graduate students (12 males and 13 females), who did not participate in the formal study and did not know the purpose of this study, were invited to rate the attractiveness of the images using scale 1 (very unattractive) through 7 (very attractive). Finally, based on the evaluation scores of attractiveness, two highly attractive faces (one male and one female) and two low attractiveness faces (one male and one female) were selected as doctoral evaluators in the formal experiment.

#### Questionnaire

##### Cheek and Buss Shyness Scale

The Revised Cheek and Buss Shyness Scale (RCBS) (Cheek, 1983) assessed shyness levels. The RCBS consists of 13 items such as “I am often uncomfortable at parties and other social functions.” Each item is rated on a 5-point scale from 1 (very uncharacteristic or untrue, strongly disagree) to 5 (very characteristic or true, strongly agree). Further, the higher the score, the higher the shyness level. This scale has high validity and reliability (Cheek and Buss, 1981; Bruch et al., 1989). The Chinese version of the RCBS has also been proven to have good reliability and validity (Xiang et al., 2018). The RCBS was administered twice: once during screening and once before the formal experiment and excluded participants who were inconsistent between screening and testing (*n* = 26). The correlation between the screening and before the formal experiment shyness data was very strong (*r* = 0.943, *p* < 0.01), and both showed excellent internal consistency in the current sample (both α = 0.94).

### Procedure

Participants were instructed to refrain from smoking, alcohol, and caffeine and get a good rest on the day before the experiment. After arriving at the laboratory, the subjects were briefed on the procedures, and informed consent was obtained. The subjects then filled out questionnaires and were fitted with an electrode cap. The EEG recordings were conducted in a dimly lit and soundproof experimental room. The participants sat quietly in a chair in front of a computer monitor. After the electrodes were attached, the resting EEG was recorded for 6-min of alternating 1-min periods of eyes-open (EO) and eyes-closed (EC) (baseline condition). The participants were instructed to relax and not move as little as possible during recording. At the end of the baseline, participants were asked to report their levels of nervousness and avoidance using an 11-point Likert scale.

Following the baseline phase, create an environment for social evaluation. The subjects were then informed that they would have to prepare a 3-min impromptu speech about their favorite or least favorite movie and explain why they liked or disliked it (Westenberg et al., 2009). Participants were told that the speech would be given in front of two Ph.D. students (one male, one female) who studied social behavior. The subjects were informed that two Ph.D. students would come in during the speech phase and give them a speech (this was not the case). Subjects were told that they could get to know the two doctors briefly. Then, we opened the photos of two doctors presented by the computer in front of the subject. One was on the left side of the computer center, and the other was on the right. Subjects were instructed that two Ph.D. students would evaluate their presentation on their strengths and weaknesses, as well as their general personalities (Kuai et al., 2020). The subjects were then told that they had 3-min to prepare their speech. To prepare the speech well, we asked them to make their speeches as close to 3-min as possible. If you stopped talking, two Ph.D. students would constantly prompt you to go on and say something more. The subjects were told that they could neither speak nor take notes and could only make imaginary constructions during the preparation time. We informed the subjects that photos of the two Ph.D. students were displayed on the computer during the preparation phase. If the photos disappeared, the preparation time was over.

The subject was left alone, and the EEG was recorded during this preparation time (anticipation condition). The high shyness and low shyness participants were randomly assigned to a high or low facial attractiveness condition before the study began. Shyness (high vs. low) and attractiveness (high vs. low) were both between-subjects factors. Subjects in the high facial attractiveness condition were presented with two highly attractive photos (one male and one female) of “doctoral evaluators.” In comparison, those in the low facial attractiveness condition showed two low attractiveness photos (one male and one female). Finally, there were 42 subjects in the high shyness condition (23 high facial attractiveness, 19 low facial attractiveness) and 52 subjects in the low shyness condition (26 high facial attractiveness, 26 low facial attractiveness). At the end of anticipation, participants rated their levels of nervousness and approach motivation again using an 11-point Likert scale.

Finally, the participants were asked to rate the two “doctoral evaluators” facial attractiveness using a 7-point Likert scale ranging from 1 (very unattractive) to 7 (very attractive) by pressing the appropriate key on the keyboard. After all procedures, we explained to the subjects that the study was just to record their state of preparing for the speech. They didn’t have to actually give a speech. We apologized to the subjects and got their understanding. Finally, we express our thanks to the participants. The study was reviewed and approved by the ethics committee of the School of Education, Shanghai Normal University.

At the end of baseline and anticipation, subjects were asked to indicate how nervous they were on an 11-point Likert scale ranging from 0 (not at all) to 10 (extremely). Further, they were asked to indicate how much they felt like doing the next part of the experiment on an 11-point Likert scale ranging from 0 (not at all) to 10 (extremely). The second question was used to indirectly measure of avoidance, because it might be not ethical to ask participants whether they wanted to avoid a situation and did nothing about it (Harrewijn et al., 2016; Poppelaars et al., 2018).

### Electroencephalogram Recording and Reduction

#### Electroencephalogram Recording

EEG data were recorded using an electrode cap with 64 Ag-AgCl electrodes (NeuroScan Inc., EI Paso, Texas, United States). Electrodes were positioned per the International 10/20 Electrode System, with a reference at left mastoid (M1) and a ground electrode located between FPz and Fz. The EEG data were also acquired from the right mastoid (M2). The horizontal electrooculogram (EOG) was recorded using electrodes placed at the external canthi of each eye. The vertical EOG was recorded using electrodes placed above and below the left eye. Further, the EEG signals were amplified using SynAmps amplifiers. The band-pass filter was set at DC-100 Hz. Furthermore, data from each channel were sampled at a rate of 1,000 Hz. An appropriate amount of electrolyte gel was applied to each electrode to increase the conduction. The electrode impedance level of each channel was kept below 5 kΩ.

#### Electroencephalogram Data Reduction

The EEG data were prepared and processed offline using MATLAB R2013a (The MathWorks, Inc., Natick, United States) and an open-source EEGLAB 12.02.6b toolbox based on MATLAB.^1^ The data sampling rate remains unchanged without downsampling. Continuous EEG data were inspected manually, one channel was judged to be a bad channel if it was with excessively noisy signals and interpolated (Kam et al., 2019). There were 31 bad channels in all subjects, including 13 in the high shyness group and 18 in the low shyness group. There was no significant difference on the bad channels between high shyness and low shyness group χ^2^(1) = 0.09, *p* = 0.76. Continuous EEG data were filtered using a 0.05 high-pass and 35 Hz low-pass filter, respectively (Fries et al., 2008; Harrewijn et al., 2016), and then re-referenced to the average mastoid signals of M1 and M2. Continuous data were segmented according to eyes-open (EO), eyes-closed (EC) (Baseline), and anticipation conditions, each containing 3-min of data. Eye movements and blinks were removed from the data using independent component analysis (ICA) for each portion. The data were then visually inspected for any remaining artifacts. If an artifact occurred in one of channels, the epoch for all channels was removed to ensure that the remaining data were identical for all sites in time (Schutter et al., 2006; van Peer et al., 2008). A fast Fourier transform with a Hanning window of 2 s width, and 75% overlap was used to estimate the spectral power (μV2) in the delta (1–3 Hz) and beta (14–30 Hz) band frequencies for the frontal, central, and parietal electrode sites separately for the eyes-open (EO), eyes-closed (EC), and anticipation conditions. Some previous studies suggested that the frontal delta-beta correlation may be an electrophysiological correlate of social anxiety (Miskovic et al., 2011; Harrewijn et al., 2016, 2018). To obtain an overall measure for frontal activity and then average power values across electrodes. Correspondingly, the power values across the electrodes of the central and parietal were averaged. The power values of FP1, FPz, FP2, AF3, AF4, F3, Fz, and F4 were averaged to obtain the composite frontal delta and frontal beta power values. The power values of C1, C2, C3, Cz, and C4 were averaged to obtain the composite central delta and central beta power values. The power values of P1, P2, P3, Pz, and P4 were averaged to obtain the composite parietal delta and parietal beta power values. A natural log (ln) transformation was performed on the mean power values to reduce skewness. The EEG composite measurements of delta and beta power were obtained by averaging the power in the EO and EC conditions under baseline conditions.

### Data Analysis

For the self-report data, non-parametric Mann-Whitney *U*-tests were conducted to analyze the difference in nervousness and avoidance between high and low shyness during baseline and anticipation conditions. This is because these variables are not normally distributed. We used a Bonferroni correction (α = 0.025) to correct the multiple comparisons.

Pearson correlation coefficients were calculated for cross-frequency coupling between ln delta and ln beta band power separately for high shyness group and low shyness group in each condition and electrode region. Because the sampling distribution of the correlation coefficients is highly skewed, it is difficult to compare the differences between the two groups in the delta-beta correlation. We used Fisher’s r to Z transformation to normalize the distribution of correlations, which allows us to use the independent-groups *Z*-tests. Then, independent-group *Z*-tests were conducted to compare the difference between high and low shyness group in the correlation. In addition, we explored the effect of facial attractiveness of evaluators (two Ph.D. students) on shyness in public speaking. Independent-group *Z*-tests were conducted to compare the difference between high facial attractiveness and low facial attractiveness group in delta-beta correlation separately for the high and low shyness groups in the anticipation condition. We elected to use a between-subjects measure of delta-beta correlation so that we were able to directly compare our findings with the majority of previous studies on social anxiety (Miskovic et al., 2010, 2011; Harrewijn et al., 2016).

In order to determine the differences between high shyness and low shyness group, and between high facial attractiveness and low facial attractiveness in absolute delta and beta power, the Electrode location (Frontal, Central and Parietal) × Condition (Baseline, Speech Anticipation) × Shyness (High vs. Low) × Attractiveness (High vs. Low) mixed factorial repeated measures analyses of variance (ANOVA) were conducted on the absolute delta and beta power.

For facial attractiveness scores, in order to determine the difference in attractiveness between the high and low facial attractiveness groups for high and low shyness. A Shyness (High vs. Low) × Attractiveness (High vs. Low) ANOVA was conducted on the facial attractiveness scores. Two-tailed tests were used for all analyses, and the level of significance was set at 0.05.

The effect sizes of all significant mean difference tests between groups were calculated. For the r and q index, Cohen (1988) proposed the following categories of explanation: < 0.1: no effect; 0.1–0.3: small effect; 0.3–0.5: intermediate effect; > 0.5: Large effect. According to Ferguson (2009), η^2^ values of 0.04, 0.25, and 0.64 in our study were regarded as the minimum, medium and strong effect sizes, respectively. The values of these indices are used to explain their magnitude.

## Results

### Self-Reported Data

**FIGURE 1:**
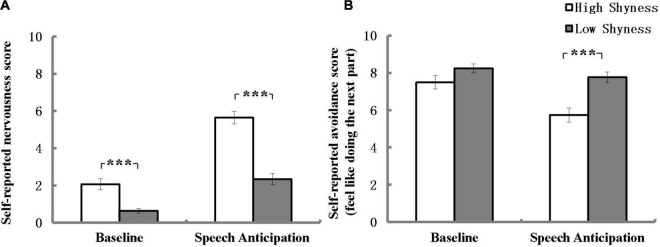
Self-reported nervousness **(A)** and avoidance **(B)** scores for high and low shyness at the end of baseline and speech anticipation. ***p < 0.001; error bars represent standard errors of the mean.

Self-reported nervousness score of high and low shyness at the end of each task are listed in Figure 1A. After Bonferroni correction (α = 0.025), as expected, the results revealed that high shyness group was associated with more significant nervousness than the low shyness group at anticipation and the effect size was large (*U* = 328.00, *Z* = –5.86, *p* < 0.001, *r* = 0.60) according to Cohen (1988). However, the results also showed that high shyness group was associated with more significant nervousness than the low shyness group at baseline and the effect size was intermediate (*U* = 636.50, *Z* = –3.72, *p* < 0.001, *r* = 0.38).

Self-reported avoidance scores of high and low shyness group at the end of each task period are listed in Figure 1B. After Bonferroni correction (α = 0.025), as expected, no difference was found in avoidance scores between the high and low shyness groups at baseline (*U* = 913.50, *Z* = –1.39, *P* = 0.17). Additionally, the results revealed that high shyness group indicated a less likelihood of participating in the next part of the experiment than low shyness group at the end of anticipation and the effect size was within the intermediate range (*U* = 578.50, *Z* = –3.95, *p* < 0.001, *r* = 0.41).

### Delta-Beta Correlation

#### Differences Between High Shyness and Low Shyness in Delta-Beta Correlation

**FIGURE 2:**
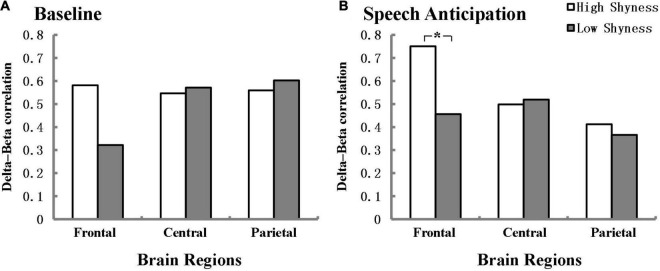
Delta-beta correlation for the high-shyness and low-shyness groups at baseline **(A)** and speech anticipation **(B)** separately for frontal, central, and parietal regions. *p < 0.05. Fisher’s r-to-Z transformation was used to normalize the distribution of correlation in the analysis.

Figure 2 illustrates the delta-beta correlations for the high shyness and low shyness groups at baseline and anticipation separately for the frontal, central, and parietal regions. In line with our expectations, high and low shyness groups have a significant difference in the delta-beta correlation for the frontal region during the speech anticipation phase and the effect size was within the intermediate range according to Cohen (1988) (*Z* = 2.25, *p* = 0.024, *q* = 0.48). The delta-beta correlation was stronger for high shyness group (*r* = 0.751, *p* < 0.001) than for low shyness (*r* = 0.456, *p* = 0.001). There was no significant difference between high shyness group (*r* = 0.581, *p* < 0.001) and low shyness group (*r* = 0.322, *p* = 0.02) participants in the delta-beta correlation during baseline in the frontal region (*Z* = 1.54, *p* = 0.12). Additionally, no differences were found between high and low shyness in the delta-beta correlation in the central and parietal regions, neither at baseline nor anticipation.

#### Effect of Facial Attractiveness on the Delta-Beta Correlation of Shyness During Speech Anticipation

**FIGURE 3:**
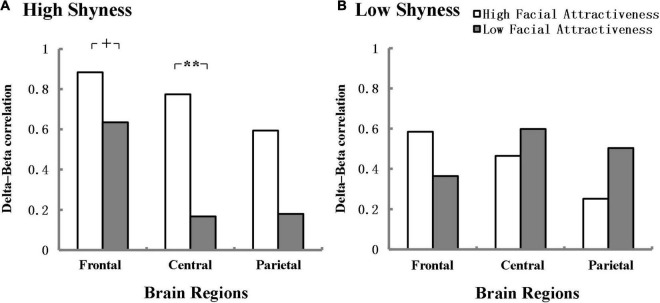
Delta-beta correlations in the frontal, central, and parietal regions for high facial attractiveness and low facial attractiveness groups at anticipation condition separately for high **(A)** and low **(B)** shyness group. ^+^*p* < 0.06, **p < 0.01. Fisher’s r-to-Z transformation was used to normalize the distribution of correlation in the analysis.

We further explored the effect of facial attractiveness on the delta-beta correlation of shyness during the speech anticipation phase. The results showed that in the high shyness group, there was a marginal difference in the delta-beta correlation between high and low facial attractiveness in the frontal region and the effect size was large (*Z* = 1.92, *p* = 0.055, *q* = 0.64), and there was a significant difference between high and low facial attractiveness in the central region and the effect size was also large according to Cohen (1988) (*Z* = 2.57, *p* = 0.01, *q* = 0.86). The delta-beta correlation was stronger for high facial attractiveness (*r* = 0.884, *p* < 0.001; *r* = 0.774, *p* < 0.001) than for low facial attractiveness (*r* = 0.635, *p* = 0.003; *r* = 0.167, *p* = 0.495). However, no significant difference was found between high facial attractiveness and low facial attractiveness in the parietal region in the high shyness group. No significant differences were found in the low shyness group. Figure 3 shows delta-beta correlations for high facial attractiveness and low facial attractiveness groups in the frontal, central, and parietal regions in the anticipation condition separately for the high and low shyness groups.

### Electroencephalogram Delta and Beta Power

**FIGURE 4:**
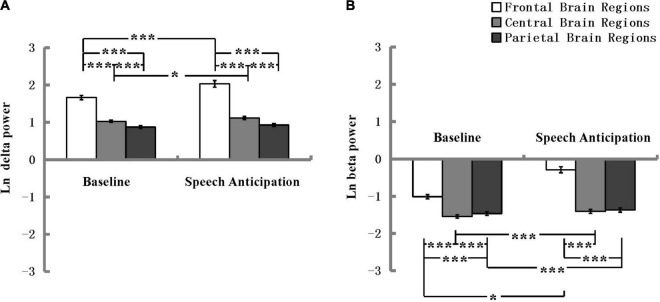
Delta **(A)** and beta **(B)** spectral power in the frontal, central, and parietal regions at the baseline and speech anticipation condition. EEG power values are natural log-transformed. *p < 0.05, ***p < 0.001; error bars represent standard errors of the mean.

For the absolute delta power, the ANOVA revealed a significant main effect of Electrode location and the effect size was strong according to Ferguson (2009), *F*(2, 180) = 379.90, *p* < 0.001, η^2^ = 0.81. The main effect of Condition was also significant and the effect size was minimum, *F*(1, 90) = 12.22, *p* = 0.001, η^2^ = 0.12. The ANOVA also revealed a significant interaction effect between Electrode location and Condition, as shown in Figure 4A, and the effect size was minimum, *F*(2, 180) = 17.04, *p* < 0.001, η^2^ = 0.16. A *post hoc* analysis revealed that delta power in the speech anticipation condition was more pronounced than that in the baseline condition at the frontal electrode location and the effect size was minimum, *F*(1, 90) = 16.73, *p* < 0.001, η^2^ = 0.16, and at the central electrode location and the effect size was minimum, *F*(1, 90) = 5.58, *p* = 0.02, η^2^ = 0.06. The absolute delta power in the frontal was more pronounced and the effect size was strong during baseline condition, *F*(2, 89) = 158.55, *p* < 0.001, η^2^ = 0.78, and speech anticipation condition and the effect size was strong, *F*(2, 89) = 113.61, *p* < 0.001, η^2^ = 0.72. There were no other significant main or interaction effects.

For the absolute beta power, the ANOVA revealed a significant main effect of Electrode location and the effect size was strong according to Ferguson (2009), *F*(2, 180) = 280.62, *p* < 0.001, η^2^ = 0.76. The main effect of Condition was also significant and the effect size was medium, *F*(1, 90) = 59.96, *p* < 0.001, η^2^ = 0.40. The ANOVA also revealed a significant interaction effect between Electrode location and Condition, as shown in Figure 4B, and the effect size was medium, *F*(2, 180) = 112.16, *p* < 0.001, η^2^ = 0.56. A *post hoc* analysis revealed that beta power in the speech anticipation condition was more pronounced than that in the baseline condition at the frontal electrode location and the effect size was medium, *F*(1, 90) = 122.91, *p* < 0.001, η^2^ = 0.58 at the central electrode location and the effect size was minimum, *F*(1, 90) = 12.77, *p* = 0.001, η^2^ = 0.12, and at the parietal electrode location and the effect size was minimum, *F*(1, 90) = 5.60, *p* = 0.020, η^2^ = 0.059. The absolute beta power in the frontal was more pronounced and the effect size were strong during baseline condition, *F*(2, 89) = 156.71, *p* < 0.001, η^2^ = 0.78, and speech anticipation condition *F*(2, 89) = 187.65, *p* < 0.001, η^2^ = 0.81. There were no other significant main or interaction effects. That is, there was no significant difference between high shyness and low shyness, and between high facial attractiveness and low facial attractiveness in either absolute delta or beta power. Table 1 shows the absolute delta and beta spectral power under different conditions and electrode site regions across the high and low shyness groups and high facial attractiveness and low facial attractiveness groups.

**TABLE 1:** Mean (SE) delta, beta spectral power across high and low shyness groups, high and low facial attractiveness groups, baseline and speech anticipation conditions, and frontal, central, and parietal electrode site regions.

EEG measures	Baseline				Speech anticipation			
	High shyness		Low shyness		High shyness		Low shyness	
	High facial attractiveness	Low facial attractiveness	High facial attractiveness	Low facial attractiveness	High facial attractiveness	Low facial attractiveness	High facial attractiveness	Low facial attractiveness
Frontal delta	1.65 (0.10)	1.77 (0.14)	1.60 (0.10)	1.64 (0.12)	1.80 (0.16)	2.20 (0.22)	1.99 (0.16)	2.14 (0.18)
Frontal beta	–0.98 (0.09)	–0.99 (0.11)	–1.02 (0.11)	–1.05 (0.11)	–0.41 (0.17)	–0.23 (0.18)	–0.17 (0.16)	–0.34 (0.14)
Central delta	1.03 (0.06)	1.09 (0.07)	0.98 (0.07)	1.01 (0.07)	1.04 (0.09)	1.24 (0.10)	1.05 (0.07)	1.13 (0.07)
Central beta	–1.52 (0.08)	–1.58 (0.10)	–1.55 (0.10)	–1.52 (0.08)	–1.51 (0.11)	–1.40 (0.12)	–1.33 (0.10)	–1.39 (0.09)
Parietal delta	0.93 (0.07)	0.91 (0.08)	0.82 (0.08)	0.84 (0.06)	0.91 (0.09)	1.03 (0.09)	0.85 (0.07)	0.92 (0.06)
Parietal beta	–1.44 (0.09)	–1.53 (0.11)	–1.48 (0.11)	–1.42 (0.09)	–1.49 (0.11)	–1.39 (0.13)	–1.28 (0.11)	–1.33 (0.11)

*EEG power values are natural log-transformed. SE, standard errors.*

### Facial Attractiveness Scores

**FIGURE 5:**
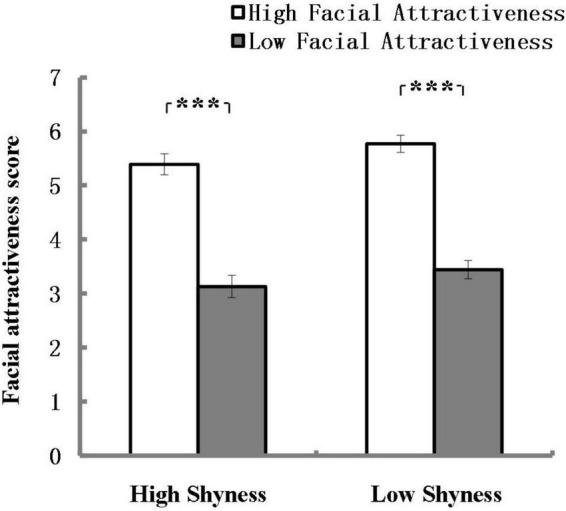
Mean of facial attractiveness scores assessed by high and low shyness participants. ***p < 0.001; error bars represent standard errors of the mean.

Means and standard errors of facial attractiveness scores assessed by high and low shyness participants are shown in Figure 5. The analysis revealed a significant main effect of Attractiveness and the effect size was strong according to Ferguson (2009), *F*(1, 90) = 160.49, *p* < 0.001, η^2^ = 0.64. Both high shyness subjects and low shyness subjects rated the high attractiveness photos with higher scores than the low attractiveness photos. There were no other significant main or interaction effects. This result validates the previous grouping of attractiveness.

## Discussion

The purpose of this study was to reveal the neural basis of shyness by delta-beta correlation and to explore the effect of evaluators’ facial attractiveness on the delta-beta correlation of shyness during the speech anticipation phase. We recorded electrical brain activity during the baseline and anticipation of public speaking. The results showed that, as predicted, the delta-beta correlation in the frontal region was stronger for high shyness than for low shyness during the speech anticipation phase. However, no significant differences were observed in the delta-beta correlation between high shyness and low shyness participants neither in the central nor parietal regions during anticipation or the baseline phase. Self-reported nervousness and avoidance were almost parallel to the differences in the delta-beta coupling correlation. High shyness was associated with more significant nervousness and felt less likely to participate in the next part of the experiment than low shyness at the end of anticipation. However, as unexpected, high shyness was associated with more significant nervousness than low shyness, not only at the end of anticipation but also at the baseline. Finally, we explored the effect of the facial attractiveness of evaluators on the delta-beta correlation of shyness during the speech anticipation phase. The results showed that the delta-beta correlation was stronger for high facial attractiveness than for low facial attractiveness in the high shyness group in the frontal and central regions. However, no significant difference was found between high facial attractiveness and low facial attractiveness in the low shyness group. Moreover, the participants were asked to rate the facial attractiveness of the two “doctoral evaluators,” and the results validated the previous grouping of attractiveness. Participants rated the high attractiveness photos with higher scores than the low attractiveness photos. This study reveals the neural basis of shy individuals through delta-beta correlation, which extends the electroencephalogram (EEG) studies of shyness focusing primarily on the hemispheric asymmetry of alpha spectral power. This suggested that delta-beta correlation might be another way to measure shyness besides alpha asymmetry.

No significant differences were found in the delta-beta correlation between participants with high and low shyness during the baseline phase in the current study. This is consistent with the hypothesis and previous studies with high vs. low socially anxious participants (Miskovic et al., 2010; Harrewijn et al., 2016; Poppelaars et al., 2018) and with (sub) clinical SAD vs. without (sub) clinical SAD participants (Harrewijn et al., 2018). The delta-beta correlation has been proven to be more promising when socially anxious participants anticipate social stress (e.g., Harrewijn et al., 2016). However, Miskovic et al. (2011) reported that patients with SAD showed a significant decrease in positive delta–beta correlation after cognitive-behavioral therapy during resting conditions. On the one hand, SAD showed differences before and after treatment in the resting state, possibly due to treatment. Moreover, intra-subject experimental design can better control individual differences and more easily obtain significant effects than inter-subject design. On the other hand, shyness and SAD may differ qualitatively (Heiser et al., 2003; Poole et al., 2017). Additionally, Poole and Schmidt (2020) found that positive shy children had a higher frontal positive delta-beta correlation than non-positive shy and low-shy children. The present study did not classify shyness and referred to general conceptual shyness which may be the reason for the inconsistent result. Moreover, as unexpected, high shyness was associated with more significant nervousness than low shyness at the baseline, but no significant difference was found in the delta-beta correlation. These findings suggest that a certain level of stress might be needed to induce anxiety to measure the delta-beta correlation of shyness. In all, caution should be exercised when interpreting and generalizing these findings because of the diversity of shyness.

One of the main characteristics of shyness is the extensive fear of being evaluated by others, resulting in avoidance and anxiety (Poole et al., 2017). The results showed that high shyness and low shyness significantly differ in the delta-beta correlation for the frontal region during the anticipation phase. The delta-beta correlation for high shyness was more substantial than that for low shyness. This was accompanied by subjective reporting that high shyness was associated with more significant nervousness and less likelihood to participate in the next part of the experiment. The results are in line with those observed previously, with the (sub) clinical SAD participants showing a stronger negative delta-beta correlation in the frontal region than participants without (sub) clinical SAD during anticipation (Harrewijn et al., 2018), with high socially anxious participants showing an enhanced negative delta-beta correlation in the frontal region as compared to low socially anxious participants during anticipation (Harrewijn et al., 2016). with finding of Miskovic et al. (2010) who found an enhanced negative delta-beta correlation in the frontal region as high compared to low socially anxious individuals during anticipation (Miskovic et al., 2010; Harrewijn et al., 2016). They found that (sub) clinical SAD and high socially anxious participants were separately accompanied by more significant nervousness and avoidance at the end of anticipation speech (Harrewijn et al., 2016, 2018). In this study, the difference between participants with high and low shyness in the delta-beta correlation was mainly in the frontal sites. This is consistent with the findings of Harrewijn et al. (2016, 2018). The correlation between the fast-wave (FW) and slow-wave (SW) oscillations of the frontal lobe reflects the cross-talk between cortical and subcortical brain regions (Schutter et al., 2006). Thus, the enhanced delta-beta correlation suggests a stronger functional coherence between cortical and subcortical regions, which increases with increased anxiety levels (Phelps et al., 2016; Poole et al., 2020). Extended results showed that no significant differences were found in absolute delta and beta power. This may mean that the increase in correlation cannot be attributed to a general increase or decrease in nervous system activity behind these oscillations and suggests that only the consistency between underlying system activity is affected (van Peer et al., 2008). This also implies that the high and low shyness groups differ significantly only in the synchronization of the delta and beta oscillations. These results reveal the neural basis of shyness through the delta-beta correlation. Moreover, resting and anticipation state results suggest that the delta-beta correlation may be a measure of shyness under specific stresses. The present findings are generally consistent with previous studies on social anxiety and support for a view that delta-beta correlation seems more promising during the anticipation of a socially stressful situation than in the resting state.

To the best of our knowledge, the current study was the first to explore the effect of the facial attractiveness of evaluators on the delta-beta correlation among shyness individuals during the speech anticipation phase. Shy people have negative self-evaluations and increased anxiety and avoidance in social situations (Carducci and Conkright, 2020). Previous studies suggested that comparing oneself with others who possess more positive characteristics might exacerbate negative self-evaluations associated with anxiety mood (Antony et al., 2005). Because people always have positive stereotypes about attractive people (Langlois et al., 2000; Liang et al., 2010). So we speculate that high facial attractiveness might increase the negative self-evaluations of shy individuals and increase anxiety in social situations. As expected, the results showed a difference in the delta-beta correlation between high and low facial attractiveness in the high shyness group in the frontal (marginally) and central regions. However, no significant difference was found in the low shyness group. This suggests that high shyness activates a stronger delta-beta correlation when faced with evaluators with high facial attractiveness. The results suggest that high facial attractiveness might increases the adverse effects of shy individuals under the threat of social evaluation. It is indirectly inferred that shy individuals are even shyer when confronted with a group of people they consider superior to them. However, we should be cautious in interpreting these results and further studies are needed. Furthermore, the study found differences between high attractiveness and ground attractiveness in high shyness in the frontal (marginally) and central regions. This is different from previous studies on social anxiety, where the main difference was mainly in the frontal region (Harrewijn et al., 2016, 2018). This coupling may begin in the frontal region and spread by volume conduction to the central and posterior (Olejniczak, 2006). The findings demonstrate that delta-beta coupling is sensitive to external influences and is consistent with previous observations (Knyazev et al., 2006; De Pascalis et al., 2020). That is, high facial attractiveness activated a stronger delta-beta correlation and was most magnitude in the frontal region, although differences were also found in the center region.

The present study has several limitations that should be interpreted with caution and addressed in future studies. First, this study did not further classify shyness but referred to general conceptual shyness, which may lead to a new result. Poole and Schmidt (2020) found that positive shy children had a higher frontal positive delta-beta correlation than non-positive shy and low shy children. Future work may be needed further to investigate the neural basis of different types of shyness. Second, we did not record EEG data during the speech task and task recovery period or other situations (such as when there was no social evaluation threat). Therefore, different results from this study may be found in other scenarios. Future work is needed to explore further the effect of facial attractiveness on shyness in other settings. Third, the exact functional significance of the delta-beta correlation remains unclear (Canolty and Knight, 2010). There is debate about whether delta power stems solely from subcortical regions (Harmony, 2013; Blaeser et al., 2017). Therefore, this needs to be interpreted with caution. Fourth, sample of the study is not very large, especially when looking at high vs. low attractive faces. Although the study found some interesting results, it should also be interpreted with caution. Finally, we used a between-subjects measure of delta-beta correlation to compare the findings with the majority of previous studies using the same measure in social anxiety (Miskovic et al., 2010, 2011; Harrewijn et al., 2016). However, it remains relatively unclear whether the sample-based approach (between-subjects) corresponds with the individual-based (within-subjects) approach (Schutter and Knyazev, 2012; Harrewijn et al., 2016). Therefore, the interpretation of the individual level (within-subjects) should be cautious. Considering that a within-subject approach may provide further information about individual differences, we performed an exploratory analysis using a within-subject measure of delta-beta correlation (see Supplementary Data).^2^ The analysis showed that within-subject measures of delta-beta correlation was stronger for low shyness participants than for high shyness participants during the speech anticipation phase, which differs from between-subject measures. While the within-subject measures of delta-beta correlation seemed to be stronger for low facial attractiveness in high shyness during speech anticipation phase, the within-subject measures of delta-beta correlation were not at a significant level. Inconsistencies with between-subject measure when using within-subject measure warrants caution, but this is similar to some previous studies using within-subject delta-beta correlation, which showed significantly more within-subject measures of delta-beta correlation in low socially anxious individuals than high socially anxious individuals during early speech anticipation phase (Poppelaars et al., 2018). The exact reasons for the discrepancy between between-subject measures and within-subject measures are uncertain, but it may be due to the methodological differences and the functional relevance of delta-beta coupling may differ across inter- and intraindividual of processing (Anaya et al., 2021). Although there was some disagreements, both between-subject measures of delta-beta correlation and within-subject measures of delta-beta correlation might be used to measure shyness. However, researches in the future should further examine the differences and functional implications of both between-subject and within-subject measures of delta-beta correlation.

## Conclusion

This study reveals the neural basis of shy individuals through delta-beta correlation analysis during resting and anticipatory speech states. The results suggest that a stronger delta-beta correlation might be the neural basis for shy individuals. Additionally, current research suggest that high facial attractiveness might increases the adverse effects of shy individuals under the threat of social evaluation. The results showed that the delta-beta correlation was stronger for high facial attractiveness than low facial attractiveness in the high shyness group. These findings may help us understand shyness and, hopefully, ultimately help people overcome its adverse psychological effects.

## Data Availability Statement

The raw data supporting the conclusions of this article will be made available by the authors, without undue reservation.

## Ethics Statement

The studies involving human participants were reviewed and approved by the Ethics Committee of the School of Education, Shanghai Normal University. The patients/participants provided their written informed consent to participate in this study.

## Author Contributions

XX contributed to the conception and design of the study, data collection and analysis, and wrote the manuscript. BL contributed to critical revisions of the manuscript. PL contributed to revision of the manuscript. DL contributed to the supervision and revision of the manuscript. All authors contributed to the article and approved the submitted version.

## Conflict of Interest

The authors declare that the research was conducted in the absence of any commercial or financial relationships that could be construed as a potential conflict of interest.

## Publisher’s Note

All claims expressed in this article are solely those of the authors and do not necessarily represent those of their affiliated organizations, or those of the publisher, the editors and the reviewers. Any product that may be evaluated in this article, or claim that may be made by its manufacturer, is not guaranteed or endorsed by the publisher.

## Footnotes

1
https://sccn.ucsd.edu/eeglab/index.php

2 We analyzed the data in a within-subjects way, extracting delta (1–3 Hz) and beta (14–30 Hz) power for each 2 s, and then calculating the Pearson correlation between ln delta and ln beta band power for each individual participant across the epochs in each condition and electrode region. The results showed that the within-subjects measure of delta-beta correlation was stronger for low shyness participants than for high shyness during the speech anticipation condition and the effect size was minimum, *F*(1, 90) = 5.52, *p* = 0.021, η^2^ = 0.06. There was no significant difference between high shyness participants and low shyness participants in the within-subjects measure of delta-beta correlation during baseline. The within-subject measures of delta-beta correlation seemed to be stronger for low facial attractiveness in high shyness participants during speech anticipation phase, but the within-subject measures of delta-beta correlation were not at a significant level.
