# Supplementary material for: Electrophysiological Correlates of Shyness Affected by Facial Attractiveness
Source: Front Psychol. 2022 Jan 5;12:739585. doi: 10.3389/fpsyg.2021.739585 (PMC8782144; doi:10.3389/fpsyg.2021.739585)
Supplement: Supplementary file 1 [file Data_Sheet_1.docx]

**Supplementary data**

**Within-subjects delta-beta correlation**

In order to derive a within-subjects measure of delta-beta correlation, we also extracted delta (1–3 Hz) and beta (14–30 Hz) power for each 2 s, and then calculated the Pearson correlation between ln delta and ln beta band power for each individual participant across the epochs in each condition and electrode region. Electrode location (Frontal, Central and Parietal) × Condition (Baseline, Speech Anticipation) × Shyness (High vs. Low) × Attractiveness (High vs. Low) mixed factorial repeated measures analyses of variance (ANOVA) were conducted on the within-subjects measure of delta-beta correlation.

The ANOVA revealed a marginally significant main effect of Electrode location and the effect size was minimum according to Ferguson(2009), F (2, 180) =3.38, p = 0.055, η2 = 0.04. The main effect of Condition was also significant and the effect size was minimum, F (1, 90) = 9.79, p = 0.002, η2 = 0.10.

The interaction effect between Electrode location and Condition was significant, as shown in supplementary figure 1, and the effect size was minimum, F (2, 180) =13.72, p < 0.001, η2 = 0.13. A post-hoc analysis revealed that the within-subjects measure of delta-beta correlation in the baseline condition was more pronounced than that in the speech anticipation condition at the frontal electrode location and the effect size was minimum, F (1, 90) =20.78, p < 0.001, η2 = 0.19. The within-subjects measure of delta-beta correlation in the frontal was more pronounced and the effect size was minimum during baseline condition, F (2, 89) = 6.52, p = 0.002, η2 = 0.13.


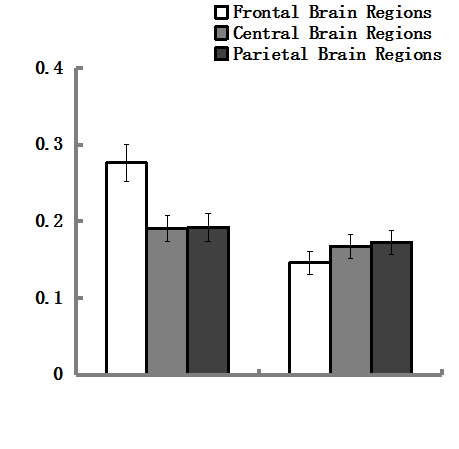


1. **Baseline Speech Anticipation**

******

*******

*****

1. **Within-subjects delta-beta correlation**

Supplementary figure 1. Within-subjects delta-beta correlation in the frontal, central and parietal regions at the baseline and speech anticipation condition. Notes: *p < 0.05, **p < 0.01, ***p < 0.001; error bars represent standard errors of the mean.

The ANOVA also revealed a significant interaction effect between Shyness and Condition, as shown supplementary figure 2, and the effect size was minimum, F (1, 90) =5.25, p = 0.024, η2 = 0.06. A post-hoc analysis revealed that the within-subjects measure of delta-beta correlation was stronger for low shyness participants than for high shyness during the speech anticipation condition and the effect size was minimum, F (1, 90) =5.52, p = 0.021, η2 = 0.06. The within-subjects measure of delta-beta correlation during baseline condition more pronounced than during the speech anticipation condition for high shyness participants, F (1, 90) = 13.22, p < 0.001, η2 = 0.13.

1. **Within-subjects delta-beta correlation**
2. **Baseline Speech Anticipation**
3. *******

*****

Supplementary figure 2. Within-subjects delta-beta correlation for the high-shyness and low-shyness groups at baseline and speech anticipation. Notes: *p < 0.05, ***p < 0.001; error bars represent standard errors of the mean.

There were no significant interaction effects between Shyness and Attractiveness at speech anticipation condition. While the within-subject measures of delta-beta correlation seemed to be stronger for low facial attractiveness in high shyness, the within-subject measures of delta-beta correlation were not at a significant level as shown in supplementary figure 3.

1. **High Shyness**
2. **Low Shyness**
3. **B**
4. **A**
5. **Within-subjects delta-beta correlation**
6. **Frontal Central Parietal**
7. **Brain Regions**
8. **B**
9. **Brain Regions**

**Frontal Central Parietal**

1. **Within-subjects delta-beta correlation**

Supplementary figure 3. Within-subjects delta-beta correlation in the frontal, central and parietal regions for high facial attractiveness and low facial attractiveness groups at speech anticipation condition separately for high (A) and low (B) shyness group. Notes: error bars represent standard errors of the mean.
